# Molecular screening of PROKR2 gene in girls with idiopathic central precocious puberty

**DOI:** 10.1186/s13052-020-00951-z

**Published:** 2021-01-07

**Authors:** Francesca Aiello, Grazia Cirillo, Alessandra Cassio, Raffaella Di Mase, Gianluca Tornese, Giuseppina R. Umano, Emanuele Miraglia del Giudice, Anna Grandone

**Affiliations:** 1 Department of Child, Woman, General and Specialized Surgery, University of Campania “L. Vanvitelli”, Naples, Italy; 2 Department of Pediatrics, University of Bologna, Bologna, Italy; 3 Pediatric Section-Department of Translational Medical Sciences, University of Naples Federico II, Naples, Italy; 4 Institute for Maternal and Child Health, IRCCS “Burlo Garofolo”, Trieste, Italy

**Keywords:** PROKR2, Early central precocious puberty, Genetic screening

## Abstract

**Background:**

Prokineticin receptor 2 (*PROKR2*) loss of function mutations have been described as cause of hypogonadotropic hypogonadism. In 2017, a first case of central precocious puberty (CPP) caused by *PROKR2* heterozygous gain of function mutation was described in a 3.5 years-old girl. No other cases have been reported yet. This study performs a molecular screening in girls with early onset CPP (breast budding before 6 years of age) to identify possible alterations in *PROKR2*.

**Methods:**

We analysed DNA of 31 girls with idiopathic CPP diagnosed via basal LH levels > 0.3 IU/L or peak-LH > 5 IU/L after stimulation, without any *MKRN3* mutations. The Fisher exact test was used to compare polymorphism allele frequency to corresponding ones in genome aggregation database (gnomAD).

**Results:**

No rare variants were identified. Five polymorphisms were found (rs6076809, rs8116897, rS3746684, rs3746682, rs3746683). All except one (i.e. rs3746682) had a minor allele frequency (MAF) similar to that reported in literature. rs3746682 presented a MAF higher than that described in the gnomAD (0.84 in our cohort vs 0.25 from gnomAD).

**Conclusions:**

As for other G protein-coupled receptors (i.e. GPR54), mutations in *PROKR2* do not seem to be a frequent cause of CPP in girls.

## Background

Idiopathic central precocious puberty (CPP) results from premature activation of hypothalamic GnRH secretion in absence of congenital or acquired organic lesions in central nervous system.

The prevalence of CPP is higher in girls than in boys [1]. Although around 70% of variation of pubertal timing seems due to genes, genetic mechanisms leading to CPP are still a field of scientific investigation [2]. So far, *MKRN3* loss-of-function mutations are the most frequently identified monogenic cause of CPP [3, 4]. Heterozygous activating mutations in *KISS1* and *KISS1R* genes have been reported as causes of few cases of CPP [5, 6]. *DLK1* loss-of-function mutations determine a more complex, yet uncommon, phenotype characterized by CPP, overweight, early onset Type 2 Diabetes, hyperlipidemia, and Polycystic Ovary Syndrome [7]. Besides that, rare cases of CPP patients primarily related to clinical syndromes or chromosomal abnormalities have been identified [8].

In 2017 Fukami et al. performed an extended molecular analysis by next generation sequencing and identified a new heterozygous frameshift mutation in *PROKR2* gene in a 3.5 years-old girl affected by CPP [9]. *PROKR2* is a G protein-coupled receptor (GPCR) expressed on the membrane of GnRH neurons, whose activation promotes GnRH secretion. Loss-of-function mutations in this gene accounts for about 5% congenital hypogonadotropic hypogonadism with or without anosmia [10]. In contrast with other mRNA frameshift mutations producing a stop-codon, the mRNA with the mutation identified by Fukami and colleagues, namely p.C242fsX305, showed to escape nonsense-mediated mRNA decay mechanism. In-vitro assay demonstrated that this variant receptor did not exert activity itself whereas boost the activity of wild-type receptor in heterozygous state. The precise mechanism of action still remains unclear. Perhaps, hyperactive mutant-wild-type heterodimers have a greater ligand affinity or a reduced receptor internalization due to the truncated C-terminal domain.

This study aimed to perform a molecular screening of *PROKR2* variations in a cohort of girls with CPP. Considering the exceptionally young age of the patient described by Fukami et al., we limited the molecular screening to girls with CPP onset before the age of 6 years.

To date, no other data of mutations in *PROKR2* in CPP are available.

## Methods

### Setting

In order to investigate the possible role of PROKR2 in the pathogenesis of CPP, a prospective, observational multicentre study was set up and carried out over a 2-year period. Four Italian centres were involved in this study: Department of Woman, Child, General and Specialized Surgery of University of Campania “Luigi Vanvitelli”, Naples, Pediatric Section-Department of Translational Medical Sciences, University of Naples Federico II, Naples, Institute for Maternal and Child Health IRCCS, “Burlo Garofolo”, Trieste, and the Department of Paediatrics, Bologna University, Bologna. Ethics committee of the University of Campania “Luigi Vanvitelli” approved the protocol then subscribed by ethics committee of the other centres. According to the World Medical Association Declaration of Helsinki, written informed consent from parents and oral consent from all participants was collected.

### Subjects

We recruited 31 females with diagnosis of CPP that met these following criteria: thelarche occurred before age of 6 years, defined as Tanner stage B ≥ 2 at physical examination, a diagnosis of central hypothalamic–pituitary–gonadal activation identified by pubertal basal luteinizing hormone (LH) levels (> 0.3 IU/l) or a positive Gn-RH stimulation test (peak-LH > 5 IU/mL) and normal brain MRI.

Exclusion criteria included: congenital defects or abnormalities possibly related to syndromic features, central nervous system pathology (i.e., tumours or nonspecific cerebral anomalies associated with CPP). All the patients involved in this study were Italian. All subjects were unrelated.

### Protocol

All girls underwent a clinical examination with special regard to auxological parameters and staging of breast development according to Tanner’s classification. Right hand and wrist X-ray were performed for bone age evaluation by TW2 method. Peripheral blood samples were collected for hormonal dosage and genetic analysis.

Chemiluminescence assay (LIAISON, Diasorin) was used to measure Follicle Stimulating Hormone (FSH) and LH concentrations, with detection limits of 0.06 and 0.05 U/L, respectively, and intra- and inter-assay CV less than 5%. Radioimmunoassay was used to measure serum estradiol (CisBio International). The analytical and functional detection limits for plasma estradiol were 4 and 8 pg/mL, respectively.

GnRH stimulation test was provided for patients in which basal hormone level did not meet diagnostic criteria for CPP. Peak-LH > 5UI/L after administration of 0.1 mg of Relefact LH-RH (Sanofi-Aventis, Frankfurt am Main, Germany) was considered positive.

Thin-section, contrast-enhanced MRI examination of sellar region with T1-weighted and T2 weighted sagittal, coronal sequences, 3DT2 thin section axial sequence and FLAIR and EPI DWI on axial sequence was acquired for all patients.

### Genetic analysis

Genomic DNA was extracted from peripheral whole blood using a DNA extraction kit (Promega, Madison WI, USA) following the manufacturer’s instructions. Each of the two coding exons and the intron-exon boundaries of the *PROKR2* gene was amplified by polymerase chain reaction (PCR) using two couples of primers each and subsequently analysed by direct sequencing (ABI PRISM 3100, Perkin Elmer, USA) under standard conditions. Primer sequences utilized in this study will be provided by authors upon request. In the attempt to reduce possible bias of selection in the sample due to other mutations, an analogous procedure was used to screen *MKRN3* gene sequence to rule out the most frequent genetic cause of CPP nowadays individuated. All genetic analyses were performed at Department of Woman, Child, General and Specialized Surgery of University of Campania “L. Vanvitelli”, Naples, Italy.

### Data analysis

Epidemiological data were expressed as medians (interquartile ranges). Fisher exact test was used to compare our polymorphisms allele frequency to those reported in gnomAD. Mann-Whitney U test or Kruskal-Wallis test were used as appropriate to compare genotype subgroups for each single-nucleotide polymorphism (SNP). Results reached statistical significance at a *p*-value less than .05. All statistical analyses were performed using Stat-Graph Centurion XVII software for Windows.

## Results

Clinical and laboratory characteristics of our cohort are shown in Table 1. Median age at first occurrence of thelarche was 5.6 (min-max: 1.1–5.9 years). Family history of precocious sexual development was identified in 26.9% of them. No mutation was found in *MKRN3* locus in the whole sample.

**Table 1:** Clinical and laboratory features of CPP girls. All continuous variables are expressed as median (IQR25-IQR75)

**Age at diagnosis,** years	6.0(5.0–6.5)
**Age at thelarche occurrence,** years	5.6 (4.2–5.8)
**PH** ≥ 2, N (**%)**	17 (54.8%)
**B > 2,** N (**%)**	12 (38.7%)
**Height SDS**	0.89 (−0.18 to 1.43)
**BMI SDS**	0.47 (−0.24 to 0.82)
**Δ bone age – chronological age,** years	2.66 (1.75–3.45)
**Basal LH,** IU/L	0.8 (0.4–2.4)
**Basal FSH,** IU/L	3.9 (3.4–5.1)
**Basal E2,** pg/mL	16 (11–27.7)
**Peak LH,** IU/L	11.3(8.2–26.7)

*PH* pubic hair Tanner stage, *N* raw numbers, *B* breast Tanner stage, *E2* estradiol

All patients were treated by GnRH analogues (triptorelin) with a good response. In particular, two patients with a breast budding occurred before 3 years of age, included in our cohort, had a very rapidly progressive form of precocious puberty, with accelerated growth, thelarche progression and in one case uterine bleeding, both requiring blocking treatment. All these clinical features excluded the alternative diagnosis of minipuberty in these patients.

No rare variants in the coding region of *PROKR2* were identified. Five polymorphisms were found as listened in Table 2. All except one patient had more than one SNPs in different combination. Four SNPs had a MAF similar to that reported in literature (Table 2). A statistically significant difference in MAF was found for SNP rs3746682: 0.84 in our cohort vs 0.25 from gnomAD (*p*-value <.0001).

**Table 2:** MAF of SNPs identified in our cohort and the corresponding one in gnomAD

SNP	Position	MAF in our cohort	MAF in gnomAD	***p***-value
**rs6076809**	c.-8-40C > T	0.06	0.03	.48
**rs8116897**	c.458 + 62G > A	0.47	0.49	.89
**rs3746684**	c.465C > T	0.37	0.40	.77
**rs3746682**	c.585G > C	0.84	0.25	<.00001
**rs3746683**	c.525C > G	0.18	0.12	.32

No differences were found for median age at puberty onset among different genotypes subgroups for each SNP (Table 3).

**Table 3:** Observed genotype frequency, expressed as raw number (rate percentage), and corresponding median age (IQR25-IQR75) for each SNP in the cohort

**rs6076809**	**CC**	**CT**	**TT**	***p*-value**
Observed genotype frequency	27 (87%)	4 (13%)	0	
Median age	5.4 (4.4–5.8)	5.8 (5.8–5.9)		.158
**rs8116897**	**GG**	**GA**	**AA**	
Observed genotype frequency	10 (32.2%)	12 (38.7%)	9 (29.1%)	
Median age	3.5 (3–5.7)	5.5 (5–5.6)	5.8 (5.6–5.9)	.212
**rs3746684**	**CC**	**CT**	**TT**	
Observed genotype frequency	11 (35.5%)	17 (54.8%)	3 (9.7%)	
Median age	3.5 (3–4.85)	5.8 (5.6–5.9)	5.4 (5.4–5.5)	.761
**rs3746683**	**CC**	**CG**	**GG**	
Observed genotype frequency	20 (64.5%)	11 (35.5%)	0	
Median age	5.8 (5.3–5.9)	5.4 (3.6–5.7)		.098
**rs3746682**	**GG**	**GC**	**CC**	
Observed genotype frequency	2 (3.2%)	7 (25.8%)	22 (71%)	
Median age	3.2 (1.5–5)	5.6 (2.9–5.9)	5 (3–5.9)	.634

## Discussion

*PROKR2* plays a critical role in regulating olfactory bulb morphogenesis and sexual maturation [11]. A hyperactive prokineticin system appears to be an obvious pathogenetic mechanism of sexual precocity. Nevertheless, no sequence rare variation was detected in the coding region of *PROKR2* in our cohort.

We do realize that our sample size is relatively small, however it is exceptionally homogenous due to strict requirements of selection: all patients had an idiopathic CPP with onset before the age of 6 and genetic analysis negative for *MKRN3*. This recruitment method allows to exclude the “grey zone” between 7 and 8 years where normal variants of “accelerated puberty/rapid progressive thelarche” can occur without medical intervention needed. In addition, it sets up a cohort with the most similar clinical features to the index case of *PROKR2* mutant precocious puberty reported by Fukama et al. Therefore, although negative, the finding of our study remains interesting. Indeed, we can speculate that if mutation in *PROKR2* coding region could explain 10% of CPP, we would have had identified at least one mutation within our sample size with a 95% level of confidence and a precision of 90%. Therefore, we can suggest that *PROK2R* is not at least a common cause for CPP, even in early onset CPP.

Those findings suggest that as for other GPCR (e.g. KISS1R) [6], gain of function variants are a very rare cause of CPP probably because hypersignal in this pathway are barely tolerated due to the critical role exerted by those genes.

Since we evaluated only coding region and intron-exon joints, we cannot rule out the presence of possible mutations outside those regions. Promoter regions and miRNAs regulatory elements should be the next target of further investigations as they may also play an important role in transcriptional and post-transcriptional control of gene expression as the experience with *MKRN3* gene in CPP has taught us [12].

We found five different SNPs in our cohort: 4 of them showed allele distribution similar to gnomAD, suggesting no individual role in CPP. The current knowledge and results of this study are insufficient to evaluate if particular combinations of polymorphisms might affect gene expression and contribute to determine CPP onset. On one side, no difference in allele frequency distribution between our cohort and gnomAD for 4 out of 5 SNPs identified and their characterization as benign variants would exclude their potential role in CPP. On the other side, epigenetic mechanism of action could be advocated, as intron variants’ role is not completely understood. Due to the small sample size and the absence of a control group, it remains unclear if particular SNPs combination might modulate the onset of CPP. Future case-control studies based on larger cohorts, are needed to investigate possible role of particular haplotype blocks in CPP.

Interestingly, we observed a statistically significant difference in MAF of rs3746682 polymorphism in our cohort compared to literature report. It is highly difficult to distinguish whether this different allele frequency has a clinical meaning or is a stochastic finding due to our small sample size. In fact, as rs3746682 (p.Thr195=) is a synonymous SNP, it probably does not display a pathological role. However, this variant has been associated with methamphetamine dependence in Japanese population [13] and bipolar disorder in Japanese females [14].

Although belonging to a different medical field, these findings suggest that this particular SNP could modulate individual’s response to certain drugs and susceptibility to develop mental disease. As CPP is a multifactorial condition, we cannot exclude rs3746682 involvement in the complex interaction between environment and genetic background. However, in absence of functional studies, it remains unclear which would be the molecular mechanism of action of this polymorphism in altering gene expression. It would be interesting to investigate rs3746682 in larger cohorts and with functional studies.

Besides, it is important to remember that murine studies on the prokineticin system demonstrated that PROK2-PROKR2 signalling seems to be implicated in many other hypothalamic functions such as the regulation of suprachiasmatic nucleus circadian clock [15]. Therefore, it is possible that mutations of this gene might cause not isolated CPP in the context of more complex phenotypes. The role of prokineticin system in the pubertal timing regulation and its disruption in humans still warrants further investigation.

## Conclusions

This study states *PROKR2* gene variants are not a common cause of CPP, also in very young girls. At the best of our knowledge, this is the first study providing a molecular screening of *PROKR2* in a selected group of patients with idiopathic CPP. Genetic aetiology of CPP remains an interesting field of research, the findings of our screening support the idea that gain-of-function mutations of genes involved in hypogonadotropic hypogonadism seem to be a very rare cause of CPP.

## Data Availability

The datasets used and analysed during the current study are available from the corresponding author on reasonable request.

## Abbreviations

CPP: Central Precocious Puberty
GnomAD: Genome Aggregation Database
MAF: Minor Allel Frequency
GPCR: G Protein-Coupled Receptor
LH: Luteinizating Hormone
FSH: Follicle Stimulating Hormone
PCR: Polymerase Chain Reaction
SNP: Single-Nucleotide Polymorphism
PH: Pubic hair Tanner stage
N: Raw numbers
B: Breast Tanner stage
E2: Estradiol
